# Architecting the Future of Research Communication: Building the Models and Analytics for an Open Access Future

**DOI:** 10.1371/journal.pbio.1001691

**Published:** 2013-10-22

**Authors:** Cameron Neylon

**Affiliations:** Public Library of Science, Cambridge, United Kingdom

## Abstract

As part of our Tenth Anniversary *PLOS Biology* Collection, PLOS' director of advocacy, Cameron Neylon, expounds on the need to improve and focus on our sharing infrastructure to maximize the reach of research communication.

We live in an exciting time. There are huge opportunities starting to open up for more effective research communication. The massive progress towards Open Access [1] is a core part of this. At the same time, the tools we have to display, manipulate, and interact with this content have become not just incredibly powerful, but easier to use. And as the web in general provides new kinds of services, new ways of communicating, telling stories, and manipulating data there is a profound cultural shift occurring as our expectations of what should be possible, indeed what should be easy, grow.

But as this vista opens up, we also have to make choices. The possibilities are multiplying but where should we focus our attention? More particularly, in a world of limited research resources where are the most important opportunities for greater efficiency? The Open Access movement has changed from a small community advocating for change, with successes in specific disciplines, to the centre of research policy making. But as we move into implementation, disagreements on details and priorities come to the surface.

These choices and the attendant disagreements are important and will occupy our attention for the next few years. But we also need to look beyond them. We need to ask ourselves what our overall priorities are for research and research communication. And we need a framework that we can use to critique the opportunities and costs that will arise as we look to extend the principles of Open Access from articles to books, grey literature, data, and materials, indeed to all the outputs of research. Increasingly researchers, institutions, and funders will be asking the question: with these resources for communication, how do I maximize the value of this research? Understanding how to answer these questions is possibly the core challenge for the next decade of scholarly communications. To meet this challenge we will need better frameworks to understand how scholarly communication works in a networked environment.

## Open Content and Open Resources

As the process of implementing Open Access accelerates, it is worth reflecting on the varied underlying arguments for it. To maximize the benefits of Open Access we must first articulate what those benefits are, which ones we are prioritizing, which are complementary, and which may pull against each other.

### The Wider Access Argument

The first and simplest argument for widening access is that the taxpaying public deserves access to the outputs of the research they fund. This is a powerful argument; one that is easy to express and one that policy makers and politicians find compelling. The argument comes in broadly three variants (See Box 1): reducing the inefficiencies and redundancies that arise when researchers themselves can't access the literature; access for the general public and taxpayer; and access for translators and public engagement specialists who help to communicate research to the wider community.

**Box 1. The Three Variants of the Access Argument**

The first and simplest argument for widening access is that the taxpaying public deserves access to the outputs of the research they fund. This argument is most effective when it concerns areas of research that are of obvious public interest: for example, medical science, environmental science, economics, as well as history, literature, and languages. This argument focuses on people, and on reading, and it places the onus of developing an understanding of the research on the user.

A variant of this line of reasoning focuses on researchers themselves, who often have limited access to research literature. Funders, institutions, and researchers see the costs in time wasted looking for information and unknowingly repeating research. Outside the academic world, governments are increasingly concerned about how the lack of access affects small and medium enterprises (SMEs), with studies suggesting that the cost in lost time and sales to SMEs is substantial [10].

A development of this argument focuses on enabling greater comprehension, either of specific issues or of science itself, and on ensuring that those who can translate, interpret, and re-use research outputs have access to them. With improved access and ability to incorporate parts of research papers in their writing, bloggers, journalists, and public information providers are better equipped to provide the layer of interpretation and synthesis that informs the wider public.

Each of these arguments tends to focuses only on access for reading. It is only when we consider the needs to interpreters and synthesizers that we see a need to enable the re-use of articles. Some argue that it is only the transmission of ideas that matter and that re-use rights are not important. I disagree with this viewpoint profoundly. The ideas may be enough for skilled interpreters in specific contexts, but permitting re-use enables a much larger group of people, and a much larger range of spaces, to aid in this synthesis. The biggest single opportunity for engaging the public with research is Wikipedia, the top hit for virtually any factual web search, and containing a set of sites that have more visitors in a day than most scholarly publishers receive in a year. Expending effort on local engagement efforts while failing to make research available and incorporated in Wikipedia will frequently be the wrong use of resources.

But all of these arguments focus on an individual user and thus they have a weakness. They don't truly recognize the benefits that arise *collectively* from the development of the Internet, where the whole is much greater than the sum of its parts. To get to the heart of the argument, and the heart of the choices we need to make, we therefore have to lift our view to the system as a whole.

### The Network Architecture Argument

The reason we are implementing Open Access today is that our information and communications architecture has profoundly changed. The Internet and the web have radically increased the number of people any given person can reach, and have reduced the costs of information transfer. In both cases the changes are by orders of magnitude. And with those changes possibilities are now in reach that simply weren't before.

Let us consider a very simple model of information diffusion. The probability that information reaches a person who will make use of it can be thought of as a function of three parameters. The first of these is the total number who would be *interested*, i.e., the number who would in an ideal world use it were they to have access. The second parameter is the proportion of those interested people who are able to find the research in the first place, i.e. the *reach* of your communication tools. Multiplying these two numbers together (the fraction that can use it times the fraction that can find it) gives you the proportion that could use the information. But we also need to divide this number by a third parameter—the *friction*, which represents the difficulty in using the information once you have it. The full calculation (see Box 2) then allows us to determine the proportion of people that actually *do* use it.

**Box 2. Proportions of Re-use**

We can express this network model with a simplified equation that gives the proportion (or probability) of re-use:
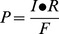
Where: *P* is the probability of information reaching a place where it can be used, or of a contribution being made to a project; *I* is the overall interest, the proportion of the population that could use the information, or could contribute; *R* is the reach of the communication method; and *F* is the friction to use, meaning how hard it is to use the information or to contribute.

The equation is an illustration—it oversimplifies a wide range of issues but is useful for seeing how even when something is difficult to use, such as raw medical literature, if there is a wide interest then by simply making it accessible the impact is significantly enhanced. It is interesting to consider what the units of the various terms might be and whether some, particularly the friction term, should have an exponent. A fully worked model would also need to include multi-step and non-linear transmission of resources to their ultimate site of application. This could likely be treated as a Hidden Markov model [11] or as a dynamic Bayesian network [12]. A full information theoretic analysis of the system is left as an exercise for the informed reader.

Let's imagine that for some piece of information the level of interest is one in a million. If the information can reach the entire world then there are about 7,000 people who could potentially use this information. As long as people can find the information easily and the friction to use it is sufficiently low, we can be confident of this work being used. On the other hand if we don't communicate effectively or if we make it difficult to use the information, it is easy to imagine that the user base would diminish rapidly.

Until 20–30 years ago the number of people we could reach was limited by the costs and logistics of print distribution. This meant that targeting was critical; finding that right few thousand people was the main focus. If targeting them increased friction for others (like paywalls) then that was a reasonable price to pay to ensure that the people we knew were most interested had the information brought to their attention.

The underlying promise of the web is that this concept is upended. The Internet, the web, and finally the read-write web have changed the number of people who can be reached from a few hundred or a few thousand to millions or even billions. With these numbers it can now be more efficient to take a scattergun approach; to reach the maximum number of people and reduce friction for all of them rather than to focus simply on targeting a few.

This is the effect that drives successful crowdsourcing, which is destroying the business model of newspapers, and which has lead to the proliferation of online communities. The level of interest in counting insects, selling through classified ads, or talking about some element of pop culture hasn't changed, but the friction has decreased—clicking a browser button is easier than joining an ecological society or getting a PhD—and the reach has increased across a threshold level that changes the nature of the system. Many of the most successful citizen science efforts gained critical mass because the story was picked up and transmitted by mainstream media—reaching beyond the community of those already engaged in a specific scientific effort.

**Figure 1:**
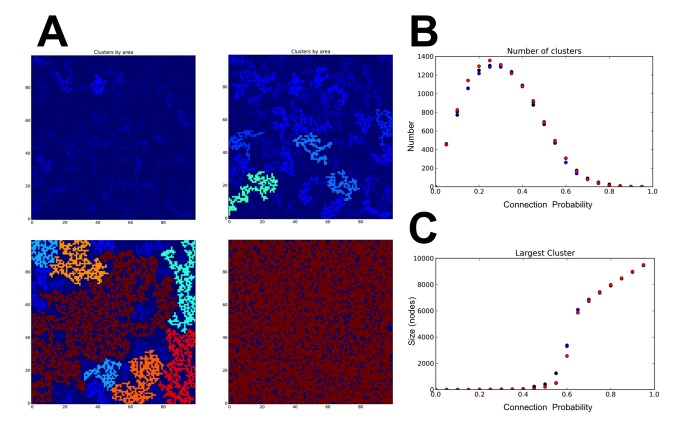
Simulations of a simple percolation network. A 100×100 matrix was created with each position on the lattice being given a random number between zero and one. To simulate a simple percolation network [13] with increasing connectivity a threshold value was raised in increments of 0.05 from zero to one. When the value at a given position was lower than the threshold value the position was considered connected to the four positions around it. The threshold value is therefore the probability of connection (or the inverse of friction). (A) Four colored plots show the size and shape of clusters at different threshold values (0.4, 0.5, 0.6, 0.8) where the cluster is colored by its size. (B) The total number of clusters for three independent simulations. The plot shows an increase in the number of small clusters to a certain level after which the number of clusters drop as they start to connect. Overall behavior is similar between three independent simulations. (C) The size of the largest cluster in the model. Up to a specific connection probability the simulation is dominated by many small clusters. At a specific probability a rapid change in size is observed as the majority of clusters connect. Behavior is highly consistent across three independent simulations. The code used to generate the figures is available at: https://gist.github.com/cameronneylon/6033364.

These shifts and changes are analogous to transitions that occur in simple networks and are easy to simulate. As the connectivity of a network increases, there is a sharp transition that occurs from a state where there are disconnected clusters to one in which most nodes are connected and a single network spans the whole system. In simple networks such as those shown in Figure 1, these transitions are highly predictable, and they occur when the probability of each point being connected (or conversely the friction) reaches a specific value. These are “disorder to order” phase transitions, similar to the crystallization of a solid from a solution. And like physical phase transitions, they occur under predictable conditions, despite the fact that the individual components of the system behave in an unpredictable fashion.

Clearly we do not live, or do science, on a simple square lattice. Yet many of the success stories of Open Research approaches and widening access seem to have some similar characteristics. In successes from the Polymath project [2],[3] to Galaxy Zoo [4], and from successful Open Source projects to Craigslist [5], a combination of scale and ease of use are the key to the story. It is also possible to look at failed efforts in crowdfunding, and in citizen science and crowdsourcing and see similar patterns. The reasons behind a lack of success can usually be traced to a failure to reach sufficient scale, which is often in turn associated with too much friction, preventing easy user interaction.

The “proportion of reuse” equation proposed in Box 2 is at best an analogy. These simple networks however show more promise as the beginnings of a model. They can provide an approach to identifying system parameters that are important in determining the system behavior. They can provide a test-bed where we can make comparisons with what we observe in our real research environment and a place where we can run experiments that we couldn't do in the real world. Models can serve different functions in the physical and the social sciences. In the former they provide quantitative predictions and a mental framework that is intended to mirror the true behavior of the system. In the latter, models are more a mode of working, a means of suggesting where to look for interesting behavior, without necessarily being expected to define an underlying truth.

The simple models shown here cannot yet have the status of a quantitative model. Nor are they predictive. They do however provide a means of understanding specific events. The successes in Open Approaches, such as Galaxy Zoo, occur because they are close enough to a transition to take advantage of it. In many cases this may have been in large part due to luck. But this does not need to be the case. If these network models are currently only interpretative, then our aspiration must surely be to make them sophisticated enough to obtain sufficient real world data and to make them predictive.

If we accept the idea that these transitions exist then the question we must ask ourselves is how do we build an architecture that makes them as large as possible, and how do we identify how to move towards them. In a world of limited resources where we have to make choices what is the *best* way to maximize the number of potential users and reduce friction? To make such choices we need data and we need frameworks for decision-making built on models with predictive and analytical power.

### The Financial Argument

The question of resources brings us to a core issue. If we are obliged to make choices about how we communicate research—if we must choose exactly what friction to reduce, and what people we will commit resources to reach—then it follows that we ought to make that choice wisely. There may be a tension between reaching more people and the financial costs that this incurs.

For Open Access to articles at least it turns out this isn't the case. Open Access provided by new publishers is cheaper than traditional subscription publishing [6],[7] and also enables research findings to reach more people, thereby facilitating their re-use. Making research available through repositories can also deliver greatly enhanced access with limited additional costs [7]. There are transitional costs involved in the shift to Open Access, particularly the issues of paying twice as revenue streams shift from subscriptions to other channels. But through careful management and a balance between the repository and journal routes the transitional costs can be minimized and massive potential downstream savings released [6],[7]. If, and it is admittedly a big if, we can transition via a blend of repository and journal based Open Access to an effective market in publication services then the transitional costs can be effectively constrained. If we get it right then we can also bring the long-term savings forward and use them to support more effective sharing.

The cost benefits that we can realize for Open Access articles depend in large part on an existing funded infrastructure, an existing *platform* for transmitting and managing these resources. But if the network argument made above holds for articles then it necessarily also applies to other kinds of research output, particularly data, but also materials.

It is interesting that some of the strongest evidence we have for the benefits of open approaches are for data, specifically the data from the human genome project where the economic returns from the publicly shared genome project were significantly greater than those from the competing closed project [8]. This success relied on an investment in the infrastructure for sharing DNA sequences, an infrastructure that is now a core part of modern biological research.

But for some other data types the platforms have yet to be created or are currently under funded. In terms of materials, platforms only exist for the sharing of very specific types. Building the right kinds of platform can increase reach, and reduce friction, but it also requires investment. The distinction between data sharing and material sharing is also not as great as it seems. And in a world where it can be cheaper to re-do an analysis than to store the data, we need to consider seriously the social, physical, and material infrastructure that might support the sharing of the material outputs of research.

Global large scale data and materials sharing is almost certainly too expensive to consider today, but we should work hard to identify the places where it can bring the greatest benefits. There will be arguments around public access, network architecture, and cost to balance and consider but with limited resources we cannot tackle the whole space immediately. But as we reap the benefit of the transition to Open Access we need to consider, as a community, where we can best apply the billions of dollars that we will liberate from subscription budgets. The key question will be how to gather the information and build the models that will help us make those choices at the system level. Without better data on how research outputs are being used we will be flying blind, but obtaining better data will also require investment.

## Mapping the Future—Foundations and Architecture

If the opportunities that we have today to re-think and mould the architecture of our communication and sharing systems are huge, then the challenges are also significant. Resources for research will continue to be flat or falling for the foreseeable future as the global economy stutters towards recovery. We will need to make difficult choices on resource allocation, and particularly to understand the balance between supporting the research itself and its communication.

These choices are not about which projects to fund or which infrastructure to build. We are bad at picking winners and show no signs of getting any better. The choices we should make are rather about how to configure the systems, how to design the processes by which we make choices, so as to optimize the overall outcome. But at the moment we have neither the models to help us do this design work, nor the data to test such models.

It is not simply, as Jeff Hammerbacher once pithily stated that “The best minds of my generation are thinking about how to make people click ads” [9]. Those “best minds” also have much better data on information flow and usage than we have in the research community. The data we have are poor and expensive, the analytics limited at best. Compare the sophistication that free tools such as Google Analytics provide in dissecting how well an advertisement with two subtly different borders performs with our ability to understand whether citations refer to the argument in a paper or the use of its data.

We have choices ahead of us, as well as opportunities to deliver significant changes in our research capacity. If we get them right. To make the right choices we need both the frameworks to help us understand the complex systems of research communication and much more data to test and utilize those frameworks. We don't just need infrastructures for sharing content and data. We need infrastructures that support the sharing of data *about* the sharing process.

Ultimately, while sharing knowledge more effectively is generally a greater public good in its own right, in the longer term it may be that significant benefits arise from our increased ability to understand how effectively that knowledge is being shared. The closed systems of the past were a necessary balance between reach, targeting, and resources. The tensions between these key issues today are entirely different to what they were in a print world. But we don't yet understand in detail how. Developing that understanding is critical to realize the full benefits of Open Access and Open Data in a resource limited world.
